# Integrative operations management framework for large-scale tertiary hospitals: a multidisciplinary empirical analysis

**DOI:** 10.1108/JHOM-07-2025-0440

**Published:** 2026-04-06

**Authors:** Jackie Zhanbiao Li, Yingqian Lao, Yuyan Shen, Saeed Awadh Bin-Nashwan, Haichang Jiang, Qiwen Jiang, Xuemei Kuang

**Affiliations:** Faculty of Business, Curtin University Malaysia, Miri, Malaysia; Affiliated Banan Hospital of Chongqing Medical University, Chongqing, China; The First Affiliated Hospital of Guilin Medical University, Guilin, China; Shanghai Putuo District Maternity and Infant Hospital, Shanghai, China; College of Commerce and Business Administration, Dhofar University, Salalah, Oman; School of Information Engineering, Jingdezhen University, Jingdezhen, China; School of Economics and Management, Southeast University, Nanjing, China

**Keywords:** Operations management framework, Tertiary public hospitals, Qualitative method, Quantitative method, Case study

## Abstract

**Purpose:**

An interdisciplinary operational management framework, which includes strategic planning, business performance and operations management groups for large tertiary public hospitals, has been proposed to address issues such as resource distribution and inefficient cross-departmental collaboration.

**Design/methodology/approach:**

Data were collected from January 2023 to December 2024 at a large hospital in Eastern China for a case study. Based on resource orchestration theory, this study thoroughly checks the application effect of the operational management framework in a case hospital by employing both qualitative and quantitative analyses.

**Findings:**

Collaboration efficiency and resource allocation had been rationalized, a 30% reduction in patient wait times and a 2.5-day decrease in hospital stays. Hospital efficiency increased from 0.72 to 0.88, enhancing patient and employee satisfaction.

**Originality/value:**

Hospital operations, patient experience and sustainable hospital development have been enhanced and supported, offering valuable insights for global hospital management practices.

## 1. Introduction

Tertiary public hospitals are central to global healthcare systems, serving multiple critical functions (Hu *et al*., 2015; Jiang *et al*., 2020). These hospitals operate in highly complex and high-pressure environments, regardless of whether they are in developed or developing countries. This is particularly evident in China's tertiary public hospitals, which face unique challenges due to their scale and scope of services (Gao *et al*., 2021; Zhu and Song, 2022). In addition to providing essential medical care, Chinese tertiary public hospitals undertake responsibilities such as medical education, scientific research and public health emergency responses, bearing significant social responsibilities (Yang *et al*., 2021; Shu *et al*., 2022). However, these hospitals encounter substantial management challenges, including high patient volumes, diverse service demands and uneven resource distribution.

The role of China's tertiary public hospitals in the global healthcare system is becoming increasingly significant. As the most populous country in the world, China relies on its tertiary public hospitals to play a critical role in safeguarding public health and responding to public health crises (Guo *et al*., 2023; Li *et al*., 2026a, b). For example, during the COVID-19 pandemic, these hospitals made substantial contributions to global efforts by efficiently allocating medical resources and providing international medical assistance (Wang *et al*., 2021). Furthermore, with the continuous advancement of medical technologies, many of China's tertiary hospitals have established themselves as global leaders in medical research and technological innovation, driving progress in global healthcare (Qiao, 2023; Li *et al*., 2025b).

However, traditional vertical management models remain predominant in many hospitals, creating significant barriers to interdepartmental collaboration and leading to inefficiencies in the flow of information and resources, ultimately impairing overall operational performance (Zhao *et al*., 2023; Li *et al*., 2024, 2025a). Addressing these management challenges has become a universal issue for tertiary public hospitals worldwide, particularly in China. To optimize hospital management and upgrade operational efficiency, there is an urgent need to establish a scientifically grounded and well-structured operations management framework.

Resource orchestration theory (ROT) emphasizes the optimization of information and resource flows, enhances the efficient allocation of unique hospital resources, and improves internal collaboration and competitive dynamics. Based on ROT, this study proposes an interdisciplinary hospital operations management framework designed to address common management challenges in tertiary hospitals. The framework focuses on optimizing interdepartmental collaboration and resource allocation to enhance overall hospital operational efficiency. It also demonstrates practical impact on strategic integration, performance enhancement and operational optimization.

The rising global significance of China's tertiary public hospitals highlights the critical need for optimizing their management models. Such optimization is essential not only for driving domestic healthcare reform but also for ensuring the efficient functioning of the global healthcare system (Cai *et al*., 2023). Therefore, establishing a comprehensive hospital operations management framework can enhance hospital efficiency, effectively address existing management issues and bridge theoretical gaps in hospital governance. Furthermore, it can contribute valuable insights to the sustainable and high-quality development of the global healthcare sector.

## 2. Theory and operations management framework

### 2.1 Theoretical foundation: resource orchestration theory and its relevance to hospital management

Hospitals, as complex social systems, face growing operational challenges, including rising patient demands, uneven resource allocation and limited cross-departmental coordination (Afilal *et al*., 2016). Increasing expectations for care quality and sustainability have elevated the importance of scientific operations management (Alboliteeh *et al*., 2023). Within this context, ROT offers a strategic framework for managing scarce resources to improve organizational performance.

Originally developed by Sirmon *et al*. (2007), ROT centers on three interrelated processes: structuring, bundling and leveraging resources. Structuring involves acquiring, accumulating and reconfiguring resources. In hospitals, this includes recruiting specialized personnel, investing in diagnostic equipment or reallocating underused resources across units. Bundling refers to the integration of resources to build capabilities. Hospitals may, for example, combine clinical expertise with digital technologies to enhance diagnostic speed or care coordination. Leveraging is the deployment of these capabilities to achieve desired outcomes, such as reducing patient wait times or improving department-wide efficiency. This requires aligning cross-functional resources with strategic objectives and ensuring cohesive interdepartmental action.

ROT's relevance in healthcare is supported by studies such as Sun *et al*. (2023) and Wang *et al*. (2018), which show how orchestrated resource management can enhance hospital outcomes, especially under resource constraints. To bridge theory and practice, Whitmore *et al*. (2024) introduced the idea of macro-operations thinking, positioning operations management as the integrative hub for strategic, clinical and managerial coordination. This systems-based perspective aligns with ROT and mitigates the limitations of rigid vertical structures through flexible collaboration.

Evaluating ROT-informed interventions requires robust implementation analysis. The RE-AIM framework – which assesses Reach, Efficacy, Adoption, Implementation and Maintenance – has proven effective in analyzing complex health system reforms (Glasgow *et al*., 1999; Nilsen, 2015). Thus, ROT provides both a conceptual foundation and actionable guidance for designing integrated hospital operations frameworks.

### 2.2 Framework design: integrated operations management framework

By integrating best practices from both domestic Chinese and international hospitals, this study proposes an integrated hospital operations management framework grounded in ROT. The framework includes an operations management structure, operational model and a detailed division of responsibilities. The operations management structure operates through a three-tiered hierarchy under the strategic oversight of the Hospital Operations Management Committee, responsible for overarching policy formulation and alignment with national healthcare objectives. Three core functional units are directly managed by the Hospital Operations Management Department, including the Strategic Planning Group, the Business Performance Group and the Operations Management Group. The Strategic Planning Group focuses on formulating strategic goals and allocating resources, while the Business Performance Group specializes in performance monitoring and data analysis. The Operations Management Group is responsible for optimizing operational processes and supporting interdepartmental collaboration. Each group has clearly defined roles, recruitment standards and task specifications to ensure scientific validity and practical implementation. In a word, this structure combines top-down strategy with cross-team collaboration to ensure efficiency and compliance.

**Figure 1:**
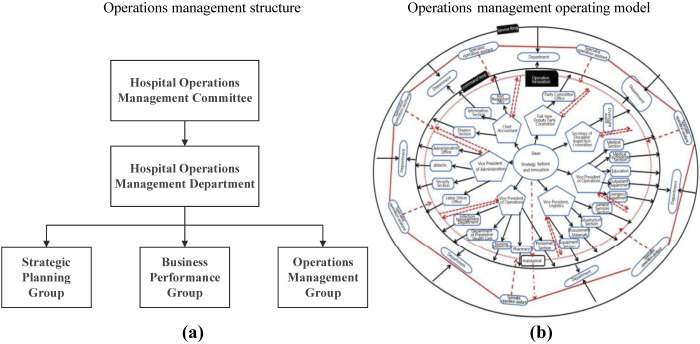
Operations management structure and operating model

Based on existing literature, the effectiveness of the integrated framework has been validated through case analysis and explores its role in promoting high-quality hospital development. The operations management structure, operational model and division of responsibilities were meticulously designed to ensure the framework's practical applicability, which (e.g. Figure 1 and Table 1) provide clear operational guidance for implementing the proposed framework.

**Table 1:** Roles and responsibilities of the operations management department

Group	Team background	Primary responsibilities
Strategic Planning Group	Members hold master's degrees or higher, with expertise in medical management, nursing, accounting, marketing, astronomy and so on	① Develop medium- to long-term strategic plans for the hospital, aligning market trends, government policies and the hospital's developmental objectives to provide strategic direction ② Design resource allocation strategies based on strategic goals and drive the implementation of critical projects ③ Conduct financial budgeting and resource coordination to ensure the achievement of strategic objectives ④ Evaluate the performance of hospital departments to ensure alignment with the overall institutional goals
Business Performance Group	Members hold master's degrees or higher, with expertise in mathematics, accounting, physics, computer science, marketing and so on	① Assess the hospital's operational performance using data analysis, financial modeling and market research, focusing on profitability, cost control and financial operations ② Analyze revenue, expenses and patient flow to provide decision-making support to management ③ Develop and rationalize hospital business management plans to improve operational efficiency and performance ④ Monitor industry dynamics and market trends to propose innovative business strategies
Operations Management Group	Members hold master's degrees or higher, with medicine, nursing, physics, chemistry, computer science and so on	① Restructure internal hospital processes, including clinical workflows, patient management and logistical support, to enhance efficiency ② Facilitate interdepartmental coordination to improve communication, resource sharing and cross-departmental collaboration ③ Promote hospital information system development, leveraging data and technology to enhance operational processes (e.g. electronic medical records, data analytics) ④ Ensure compliance with hospital operational regulations, oversee quality control systems and calibrate the quality of healthcare services

**Note(s):** To enhance transparency and replicability, we need to carefully elaborate on the key interventions embedded within the operations management model beyond structural changes. ①First, a comprehensive cross-departmental incentive scheme was introduced. This scheme combined individual and shared KPIs, including joint protocol compliance, interdepartmental response times and collaborative problem-solving metrics. These were formally integrated into the hospital's quarterly evaluation system and tied to both financial rewards and strategic resource access (e.g. staffing, diagnostic equipment). ②Second, a performance transparency mechanism was deployed via a digital dashboard accessible to all middle and senior managers. The dashboard visualized shared KPIs in real time and ranked departmental performance on collaborative indicators. This created behavioral nudges for information-sharing and mutual support. ③A third pillar involved change facilitation teams embedded within the Operations Management Group. These teams served as internal consultants to support departments in aligning their processes with the new model. They conducted process walkthroughs, facilitated alignment meetings and coached team leaders on orchestration practices. Overall, these measures, alongside leadership endorsement and iterative feedback loops, formed the hospital operational management core of the transformation, converting structural intentions into behavioral change

### 2.3 Structural innovation in hospital operations: from departmental silos to integrated governance

Prior to the implementation of the new framework, the hospital operated under a traditional vertical management structure, where departmental autonomy was high, and coordination between clinical, administrative and logistical units was limited. Decision-making was often fragmented, and performance metrics focused almost exclusively on siloed indicators.

The new model introduced a centralized operations management structure comprising three interdependent units: the Strategic Planning Group, Business Performance Group and Operations Management Group (See Figure 1 and Table 1). However, the transformation extended far beyond structural redesign. Key interventions included:

1. *“The consolidation of multiple overlapping committees into a unified Hospital Operations Management Committee to streamline decision-making.”*
2. *“Introduction of hospital-wide integrated performance dashboards to replace departmental-only KPIs, enhancing transparency and shared accountability.”*
3. *“Standardization of cross-departmental workflows through digital protocol templates and coordination checklists.”*
4. *“Implementation of biweekly interdisciplinary alignment meetings chaired by the Operations Management Group to resolve cross-functional bottlenecks.”*
5. *“Training programs for middle managers focused on collaborative leadership, resource orchestration and interdepartmental negotiation.”*

These changes redefined managerial responsibilities, eliminated redundant reporting lines and reshaped incentive alignment to focus on system-wide rather than departmental optimization. Furthermore, based on the new hospital operations management framework, model and the defined roles and responsibilities of the operations management department, hospitals must implement a combination of cross-departmental incentive mechanisms, real-time performance transparency tools and embedded change facilitation teams (See Table 1). These measures are essential for transforming structural reforms into sustainable behavioral changes, thereby enabling hospital operations management to evolve from fragmented departmental silos to an integrated governance model.

## 3. Methodology

In order to address the multidimensional complexity of hospital operations, a case study has been employed to conduct multidisciplinary empirical analysis grounded in ROT. When evaluating the impact of the operations management framework, it integrates principles from the RE-AIM framework, such as desired outcomes, long-term sustainability and other critical aspects. The synergy of multiple methods could effectively capture the effects of this operation management framework in terms of departmental collaboration, resource allocation efficiency and satisfaction. The following sections detail the research design, data collection and analysis methods, and the implementation process.

### 3.1 Case background

A large tertiary general hospital in China's eastern coastal region has been selected as the case study subject. The hospital has 2,000 beds, an annual outpatient volume of approximately 3 million visits and an annual inpatient volume of 200,000, making it one of the larger comprehensive hospitals in the region. Despite its size and wide range of services, the hospital faces several operational management challenges. Patient satisfaction remains at the industry average (85%) without significant improvement, negatively impacting the hospital's brand reputation and patient loyalty. Interdepartmental communication efficiency is low, with an average completion time of 2.5 days for cross-department tasks and approximately 15 communication errors per month, reflecting poor collaboration among departments. Financial pressures are mounting, particularly in equipment procurement and the adoption of new technologies, with a 10% rise in budget but only a 5% revenue growth rate. Clinical process indicators also exceed industry benchmarks: average patient waiting time is 45 min, inpatient stays last 8 days, and surgical queue times exceed two weeks, all of which hinder the hospital's overall development. These challenges provide a typical case background for exploring a management framework focused on interdisciplinary team collaboration.

### 3.2 Data collection

The data was collected from a large tertiary general hospital between January 2023 and December 2024. A combination of quantitative and qualitative data collection methods was employed to provide comprehensive support for the case analysis. (1) Quantitative data included three key indicators: hospital operational performance, clinical process efficiency and interdepartmental collaboration. ① Hospital operational performance indicators: These metrics encompass revenue, costs, patient flow and service quality, reflecting the hospital's overall performance. ② Clinical process efficiency indicators: These include patient waiting time, length of hospital stay and surgical queue time, used to evaluate the efficiency of medical services. ③ Interdepartmental collaboration indicators: These metrics capture the average time required to complete cross-department tasks and the frequency of communication errors, providing a quantitative measure of collaboration efficiency. (2) Qualitative data were collected through in-depth interviews and observational records. For the interviews, the research team conducted sessions with 20 management members, 30 frontline medical and logistical staff, and 386 patients to identify collaboration challenges and management needs. Each interview followed a semi-structured format with approximately 15–20 questions, each lasting 45 min on average. The interviews explored challenges in collaboration, operational bottlenecks and management needs. Observational records were collected by attending cross-departmental collaboration meetings and emergency response processes, documenting key issues and areas for improvement.

### 3.3 Analysis methods

#### 3.3.1 Complex network analysis

**Figure 2:**
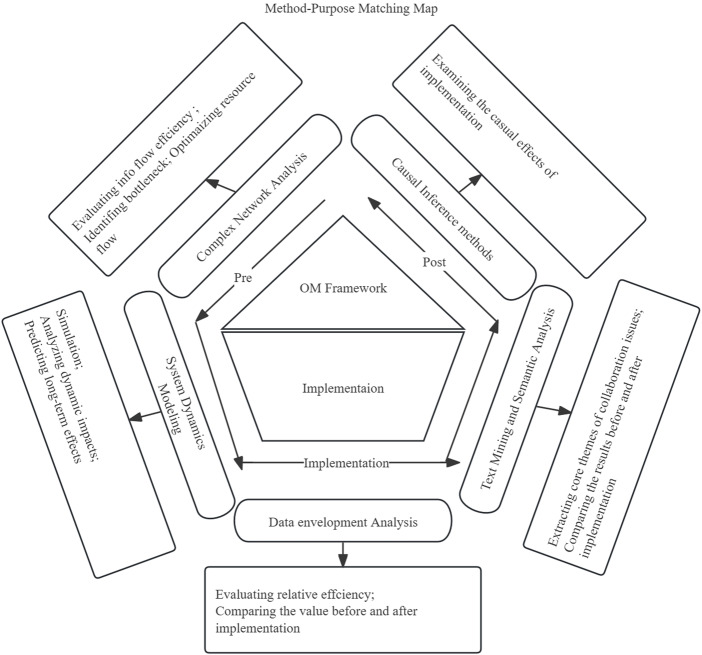
Method-purpose matching map

Complex network analysis (CNA) is composed of interconnected elements (nodes) and their relationships (edges) (Opsahl *et al*., 2010) (See Figure 2). It focuses on patterns like connectivity, centrality and community structure to understand real-world phenomena in social networks. A collaborative network diagram was constructed using Gephi 0.10.1 software to evaluate the efficiency of information flow and identify bottlenecks by calculating network density, centrality and clustering coefficients. This analysis helped assess the centrality of various departments in the hospital network and their role in the flow of information. Resource flow optimization was then designed to minimize collaboration barriers caused by information gaps and resource competition.

#### 3.3.2 System dynamics modeling

System dynamics modeling (SDM) was adopted to develop a simulation model to analyze the dynamic impacts of strategic resource allocation on hospital efficiency and predict the long-term effects of the management framework (Sterman, 2000)(See Figure 2). Feedback loops and positive feedback mechanisms were incorporated into the model to reflect complex internal interactions and enhance efficiency. The model focused on the dynamic allocation of key variables such as human resources, funding and equipment utilization rates, while assuming minor factors remain unchanged. It also assumed that the management framework would be implemented as planned during the forecast period, with historical data representing future trends. In addition, the hospital's operational environment was assumed to remain stable, with external factors' impacts being negligible. Vensim DSS 8.2.1 was used for the simulation.

#### 3.3.3 Data envelopment analysis

Data envelopment analysis (DEA) is a nonparametric method for evaluating the efficiency of decision-making units by comparing inputs to outputs (Banker *et al.*, 1984) (see Figure 2). The relative efficiency of different departments was assessed using manpower and time as the main input variables and patient flow and financial metrics as the primary output variables. Efficiency values were compared before and after the implementation of the hospital operations management framework to assess its impact on improving departmental efficiency. MaxDEA 8 Ultra software was used for the analysis.

#### 3.3.4 Text mining and semantic analysis

Text mining and semantic analysis (TMSA) was conducted on interview and observation records to extract core themes of collaboration issues, such as information gaps and resource competition (Bazeley and Jackson, 2013) (see Figure 2). Semantic analysis, in this context, focused on interpreting meaning and conceptual relationships across stakeholder narratives. The interview participants were selected to ensure comprehensive representation across hierarchical levels, functional roles and operational contexts. Management members (*n* = 20) included senior executives such as hospital directors and department heads, ensuring insights into strategic decision-making. Frontline staff and logistical staff (*n* = 30) comprised physicians, nurses and support personnel from high-interdependence departments to capture workflow challenges and interdepartmental collaboration barriers. Patients (*n* = 386) were stratified by care type and treatment duration to reflect diverse service experiences. Ethical review ensured informed consent and balanced participation across gender, age and tenure to minimize bias. The qualitative data were encoded into four categories using NVivo 14 software: information sharing platforms, cross-department incentives, resource allocation disputes and information asymmetry. The results before and after the implementation of the operations management framework were compared to provide qualitative evidence for the design of the management framework.

#### 3.3.5 Causal inference methods

Multiple regression analysis (MRA) was employed to control for confounding variables and examine the causal effects of implementing the hospital operations management framework (Angrist and Pischke, 2009) (see Figure 2). The analysis demonstrated that after implementation, key indicators such as departmental collaboration efficiency, resource utilization efficiency, clinical pathway efficiency and satisfaction levels of both patients and employees showed significant optimization, while costs and expenses were substantially reduced. In the multiple regression analysis, confounding variables were controlled by incorporating hospital-level factors and patient-level factors. The former includes variables such as professional skill levels and work experience, which may influence operational efficiency and service quality. The variables for the latter include age, gender, disease type and severity of condition, which may affect patient satisfaction and clinical pathway efficiency. This provided effective evidence that the estimated effects of the operations management framework were isolated from external confounding factors. Meanwhile, it confirms that improvements directly resulted from the framework's implementation. Stata 18 was used for this analysis.

#### 3.3.6 Analytical techniques and software tools grounded in theory

Each analytical method was selected based on its ability to capture the complexity of hospital operations and is grounded in previous research. For instance, CNA, widely used in healthcare systems analysis (Opsahl *et al*., 2010), was applied using Gephi 0.10.1 software to assess interdepartmental information flow. SDM (Sterman, 2000) was conducted using Vensim DSS 8.2.1 to simulate feedback-based resource allocation. DEA, supported by studies such as Banker *et al*. (1984), was performed using MaxDEA 8 Ultra to evaluate departmental efficiency. TMSA were implemented using NVivo 14, aligning with best practices in qualitative healthcare research (Bazeley and Jackson, 2013). MRA for causal inference followed recommendations by Angrist and Pischke (2009), and was conducted in Stata 18. Each method was chosen not only for its analytical rigor but also for its alignment with the ROT and RE-AIM evaluation framework, ensuring coherence between theory and empirical technique.

### 3.4 Implementation process

The implementation of the framework was carried out in four phases, with periodic evaluations to maximize its effectiveness:

#### 3.4.1 Phase 1: baseline assessment

A comprehensive evaluation of the hospital's current state, which included assessing performance indicators, collaboration efficiency and resource allocation to establish baseline data, was conducted before implementing the framework.

#### 3.4.2 Phase 2: pilot testing

Three departments were selected for a pilot implementation to observe the preliminary effects of the framework. Feedback from the pilot was used to refine and rationalize the framework design.

#### 3.4.3 Phase 3: hospital-wide deployment and optimization

The framework would be rolled out across the hospital following the successful pilot. Adjustments were made based on the specific needs of each department to ensure the framework's applicability and effectiveness.

#### 3.4.4 Phase 4: post-implementation monitoring and evaluation

Continuous monitoring and evaluation, which focused on assessing the framework's impact on strategic integration, performance improvement and operational optimization, were conducted after six months of the framework's implementation. Continuous adjustments were made to enhance the overall effectiveness.

#### 3.4.5 Organizational barriers and change management strategies

During implementation, several operational and behavioral barriers emerged that required targeted mitigation strategies. In the pilot phase, resistance from middle management was prominent due to concerns about increased accountability and perceived loss of departmental autonomy. Some clinical departments exhibited reluctance to share real-time data, fearing exposure of inefficiencies. To address these challenges, the implementation team adopted a participatory approach, involving key stakeholders in co-design workshops and feedback loops. Regular communication sessions were held to build consensus and demystify the objectives of the new model. In the hospital-wide rollout phase, resource reallocation – particularly staff redistribution and equipment prioritization – generated friction, particularly in departments with historically higher autonomy. The framework's emphasis on cross-functional performance evaluation and incentive restructuring initially disrupted traditional hierarchies, requiring extended onboarding and adaptation periods. These challenges underscore the importance of change management capacity and contextual adaptation in large-scale organizational transformation.

## 4. Results

### 4.1 Department collaboration analysis

**Figure 3:**
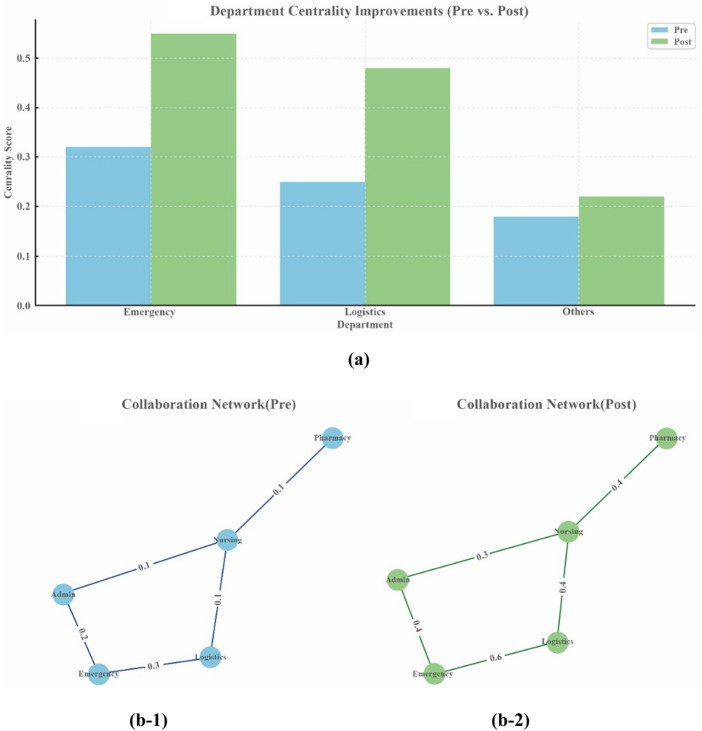
Collaboration network density

The analysis results in Figure 3 and Table 2 indicate that the implementation of the hospital operations management framework significantly enhanced interdepartmental collaboration and resource flow. The density of the departmental collaboration network climbed from 0.45 to 0.72, demonstrating a substantial enhancement in information sharing and resource mobility among departments. The Emergency Department and Logistics Department, which were identified as central hubs, showed a notable rise in centrality scores, rising from 0.32 to 0.55 and 0.25 to 0.48, respectively – improvements of 71.88 and 92.00%, both statistically significant (*p* < 0.05). The overall centrality score climbed from 0.25 to 0.44, which increased by 76%, indicating the need for comprehensive optimization of collaboration efficiency. An augmented centrality of the centralized hubs, such as the Emergency and Logistics Departments, could be observed due to prioritized resource allocation and clear task assignments, which strengthen their roles as network connectors. Upgraded resource mobility came from dynamic algorithms redirecting underused resources to high-need areas, while performance-based incentives motivated departments to share data and collaborate actively. These system-wide changes transformed fragmented operations into a unified collaborative system, leading to significant efficiency gains. These findings provide positive evidence that the hospital operations management framework effectively strengthened interdepartmental connectivity and collaboration, providing crucial support for further optimization of hospital organizational collaboration models.

**Table 2:** Enhanced department centrality improvements

Department	Pre	Post	Pre_CI	Post_CI	*p*-value	ImO (%)	Significance
Emergency	0.32	0.55	(0.28, 0.36)	(0.5, 0.6)	0.001	71.88	Significant
Logistics	0.25	0.48	(0.22, 0.28)	(0.43, 0.53)	0.003	92	Significant
Others	0.18	0.22	(0.15, 0.21)	(0.19, 0.25)	0.05	22.22	Not Significant
Total	0.25	0.44	(0.2, 0.3)	(0.37, 0.47)	0.01	76	Significant

**Note(s):** *Abbreviations:* Pre, Pre-Implementation; Post, Post-Implementation; ImO, Improvement outcomes

Especially, it is worth noting that one key mechanism supporting these improvements was the development of a cross-departmental incentive scheme that combined performance-based rewards with collaborative key performance indicators (KPIs). Departments received quarterly evaluations based not only on individual metrics such as patient throughput and cost control, but also on shared metrics such as joint protocol compliance and interdepartmental response time. These indicators were embedded in a revised incentive structure approved by hospital leadership. Incentives included both financial bonuses and access to strategic resources, such as advanced diagnostic equipment and additional staffing support. This incentive model was explicitly designed to promote information transparency, proactive collaboration and shared accountability across functional units.

Moreover, dynamic simulation modeling – conducted using Vensim DSS 8.2.1 – was critical in forecasting the long-term impact of resource reallocation under various policy scenarios. This enabled decision-makers to test incentive configurations and departmental task structures before rollout, reducing the risk of unintended consequences and supporting evidence-based implementation planning.

### 4.2 DEA efficiency valuation

**Figure 4:**
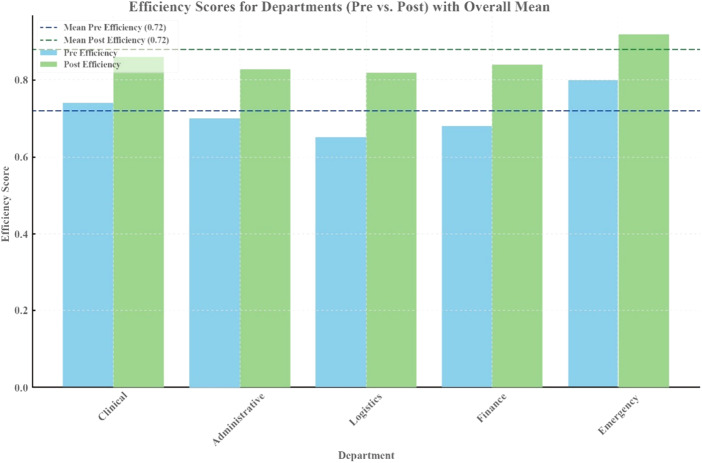
Efficiency scores for departments

The analysis results in Figure 4 and Table 3 support that the implementation of the hospital operations management framework significantly ameliorated overall departmental efficiency. The proportion of departments meeting efficiency standards increased from 68% to 85%, while the average efficiency score rose from 0.72 to 0.88. Additionally, the distribution of efficiency became more balanced with the standard deviation decreasing from 0.18 to 0.12, which indicates more equitable resource allocation. The efficiency of the Emergency Department was notable, which rose from 0.80 to 0.92, and the Logistics Department climbed from 0.65 to 0.82. In addition, these upgrades were statistically significant (*p* < 0.05). Performance incentives tied to efficiency metrics motivated departments to streamline internal workflows and collaborate proactively, driving measurable gains in both average efficiency and distribution balance. The overall efficiency improvement yielded a *p*-value of 0.0022, providing further evidence that the implementation of the hospital operations management framework has a substantial impact on enhancing operational efficiency and optimizing resource allocation.

**Table 3:** Overall efficiency scores

Phase	Mean_E	Max_E	Min_E	SD	*p*-value	Significance
Pre	0.72	0.8	0.65	0.18	–	Not Applicable
Post	0.88	0.92	0.82	0.12	0.0022	Significant

**Note(s):** *Abbreviations:* Pre, Pre-Implementation; Post, Post-Implementation; Mean_E, Mean efficiency; Max_E, Max efficiency; Min_E, Min efficiency; SD, Standard deviation

### 4.3 Operations performance indexes

**Figure 5:**
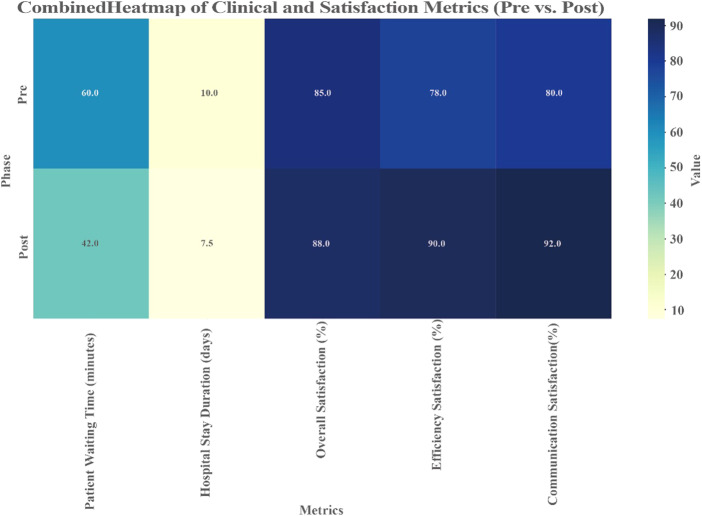
Combined heatmap of clinical and satisfaction metrics

The results presented in Figure 5 and Table 4 indicate that the implementation of the hospital operations management framework led to significant amelioration in clinical performance and patient satisfaction.

**Table 4:** Comparison of performance indicators pre- and post-implementation

	Pre	Post	Change
Clinical Efficiency Indicators			
-Patient Waiting Time	60 min	42 min	−18 min
-Length of Hospital Stays	10 days	7.5 days	−2.5 days
Patient Satisfaction Indicators			
-Satisfaction with Service Efficiency	78%	90%	+12%
-Satisfaction with communication quality	80%	92%	+12%

First, clinical efficiency indicators revealed a 30% reduction in patient waiting time, decreasing from 60 min to 42 min, and a 2.5-day reduction in the average length of hospital stays (from 10 days to 7.5 days). These results suggest more efficient service processes and a more rational allocation of medical resources, which further enhanced patient turnover rates.

Second, patient satisfaction indicators improved across the board. Overall satisfaction increased from 85% to 88%, satisfaction with service efficiency rose by 12% points (from 78% to 90%), and satisfaction with communication quality improved by 12% points (from 80% to 92%). These data demonstrate that the framework effectively enhanced the patient experience and recognition of medical services, reflecting significant optimization in service efficiency and communication quality.

Additionally, comprehensive data analysis showed that post-implementation performance metrics were more evenly distributed, and resource utilization was further optimized, highlighting the positive effects of refined management processes. This notable progress not only strengthened patient trust but also set new benchmarks for operational efficiency and service quality in healthcare institutions.

In the future, further emphasis should be placed on supporting high-demand departments, improving doctor-patient communication and dynamically monitoring key operational metrics. These steps will ensure continuous service improvement and create greater value for both patients and healthcare institutions.

### 4.4 System dynamics simulation results

**Figure 6:**
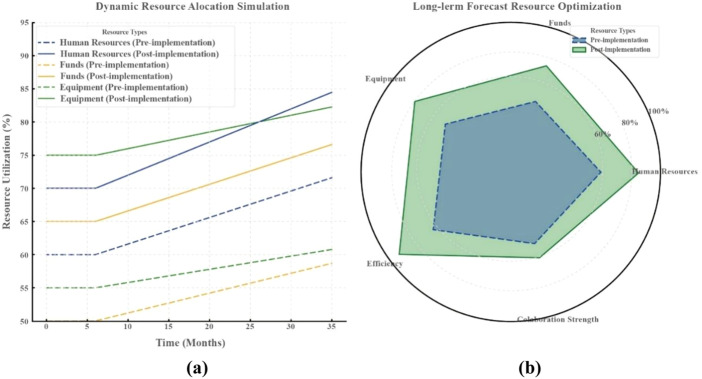
System dynamics simulation results

The analysis of Figure 6 and Table 5 demonstrates a statistically significant enhancement in the utilization rates of human resources, funding, and equipment subsequent to the implementation of the hospital operations management framework. This refinement was driven by the demand-based dynamic resource allocation, which yielded substantial short-term effects and demonstrated clear potential for long-term optimization. Specifically, human resource utilization before implementation (Pre) began at 60% and exhibited a gradual upward trend to 75% over three years. After implementation (Post), the initial utilization rate was 70%, which rapidly rose to 85% within six months before stabilizing. For funding utilization, the pre-implementation rate rose incrementally from 50% to 65% over three years, while the post-implementation rate started at 65%, reached 75% within six months and eventually reached 80% after three years. Similarly, equipment utilization moderately increased from 55% to 65% in the pre-implementation period, whereas the post-implementation rate began at 75%, climbed to 78% within six months and ultimately reached 80% over three years.

**Table 5:** Comparison of utilization indicators pre- and post-implementation

	Pre		Post (6 months)		Post (3 years)
	From	To	From	To	Eventually
Human Resource Utilization	60%	75%	70%	85%	85%
Funding Utilization	50%	65%	65%	75%	80%
Equipment Utilization	55%	65%	75%	78%	80%

These changes reflect the causal impact of the hospital operations management framework, which, through rational division of labor, resource allocation and coordination, enabled dynamic resource distribution to achieve rapid responsiveness and optimization in the short term. The initial improvements observed within six months were primarily attributed to the reallocation of resources, such as more efficient distribution of equipment and funding, which resulted in a significant marginal effect on operational efficiency.

In the long term, the steady growth in resource utilization rates indicates that the framework's dynamic adjustment mechanisms not only address short-term bottlenecks but also sustain efficiency optimization over the next three years. Projections suggest a 28% improvement in efficiency and a 10% increase in interdepartmental collaboration intensity. By implementing the hospital operations management framework, the organization achieved statistically significant enhancements in resource utilization, ameliorated overall collaborative performance and strengthened long-term sustainability.

### 4.5 Interview and text analysis results

**Figure 7:**
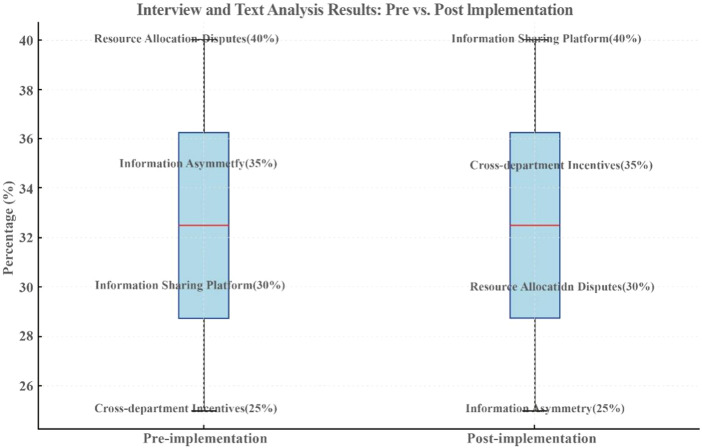
The results' differences of interview and text analysis

The interview and text analysis results presented in Figure 7 and Table 6 demonstrate significant improvements in resource allocation and information sharing following the implementation of the hospital operations management framework. Before implementation (Pre), resource allocation disputes (40%) and information asymmetry (35%) were identified as major issues, reflecting the existing challenges in resource distribution and departmental communication. Additionally, the low prevalence of cross-departmental incentive mechanisms (25%) and information-sharing platforms (30%) indicated a lack of collaboration incentives and technological support. After implementation (Post), resource allocation disputes and information asymmetry decreased to 30 and 25%, respectively, highlighting the framework's effectiveness in optimizing resource distribution and improving information flow. Meanwhile, the prevalence of cross-departmental incentive mechanisms and information-sharing platforms climbed to 35 and 40%, respectively, indicating significant enhancements in collaboration incentives and technological support.

**Table 6:** Comparison of resource allocation and information sharing pre- and post-implementation

	Pre	Post	Change
Resource Allocation Disputes	40%	30%	−10%
Information Asymmetry	35%	25%	−10%
Information-sharing Platforms	30%	40%	+10%
Cross-departmental Incentive Mechanisms	25%	35%	+10%

Overall, the implementation of the hospital operations management framework effectively addressed key operational challenges, significantly improved resource utilization efficiency and interdepartmental collaboration, and injected new momentum into hospital operations. These changes contributed to a comprehensive upgrade in both patient and employee satisfaction.

### 4.6 Causal inference methods

**Figure 8:**
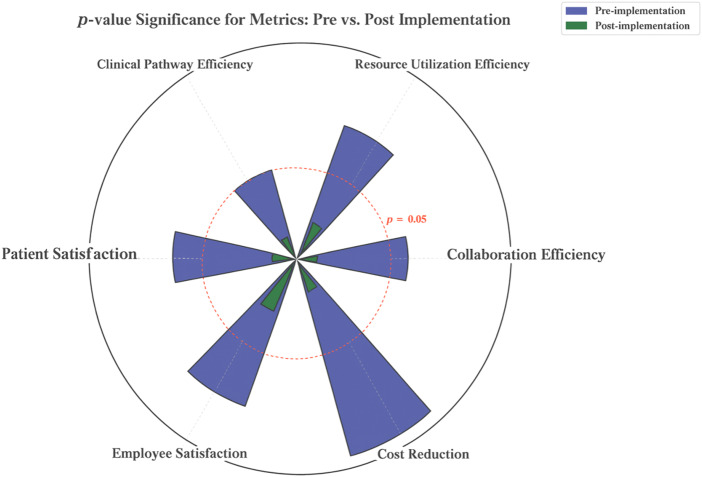
Regression analysis of causal inference methods

The multiple regression analysis presented in Figure 8 and Table 7 compares the *p*-value distributions of six KPIs at two time points: prior to (Pre) and subsequent to (Post) the implementation of the hospital operations management framework. Blue bars represent pre-implementation values, while green bars represent post-implementation values. The visualization demonstrates a clear pattern: green bars are predominantly below the red dashed line (*p* < 0.05), whereas most blue bars exceed this threshold. This statistical visualization highlights the statistically significant improvements achieved after implementing the framework. Specifically, before implementation, the *p*-value for most KPIs exceeded the significance threshold, indicating that observed enhancement lacked statistical significance: departmental collaboration efficiency (0.06), resource utilization efficiency (0.08), clinical pathway efficiency (0.05), patient satisfaction (0.07), employee satisfaction (0.09) and cost savings (0.12). This suggests that no meaningful marginal effects were detected for those indicators before the framework's implementation. Post-implementation, *p*-value for these indicators fell below the 0.05 significance threshold, several reaching a higher level of statistical significance. These results were: departmental collaboration efficiency (0.01), resource utilization efficiency (0.02), clinical pathway efficiency (0.01), patient satisfaction (0.01), employee satisfaction (0.03) and cost savings (0.02). These results confirm that the implementation of the framework yielded statistically significant improvements with measurable effect sizes t across all critical KPIs, including collaboration efficiency, resource utilization, clinical pathway efficiency, patient and employee satisfaction and cost control. This provides empirical validation of the framework's effectiveness in enhancing hospital operational and managerial performance.

**Table 7:** Comparison of *p*-value of KPIs pre- and post-implementation

	Pre	Post
Departmental Collaboration Efficiency	0.06	0.01
Resource Utilization Efficiency	0.08	0.02
Clinical Pathway Efficiency	0.05	0.01
Patient Satisfaction	0.07	0.01
Employee Satisfaction	0.09	0.03
Cost Savings	0.12	0.02

## 5. Discussion

The impact of the hospital operations management framework on improving collaboration efficiency, resource optimization and patient experience, has been analyzed and supported by empirical data validation. The findings indicate that implementing the hospital operations management framework significantly enhances overall hospital operational efficiency, particularly in improving collaboration networks, optimizing resource allocation and enhancing patient experience. Through cross-departmental incentive mechanisms, the information flow and resource integration within the hospital have been strengthened. Moreover, the common issue of information silos has been effectively addressed in traditional hospital settings, thereby enhancing hospital management capabilities. For instance, enhanced interdepartmental collaboration between the Emergency Department and the Logistics Department has directly improved managerial efficiency, augmented satisfaction levels between both patients and staff, and ultimately contributed to the high-quality development of the hospital. These findings align with recent studies suggesting that cross-departmental collaboration and information sharing can significantly enhance overall hospital operational efficiency, particularly in complex hospital management environments (Kakemam *et al*., 2020; Hübner *et al*., 2024).

Viewed through the lens of ROT, the results demonstrate how hospital performance can be strengthened by deliberately structuring, bundling and leveraging organizational resources. The centralized operations management structure enabled effective resource structuring; shared KPIs and performance dashboards facilitated capability bundling; and facilitation teams and incentive realignment mechanisms helped leverage these capabilities toward strategic outcomes.

This case reinforces that successful hospital operations management depends not only on redesigning organizational structures, but also on orchestrating human, informational and technological resources to foster system-wide alignment. From this, three practical principles emerge: (1) embed orchestration roles into formal governance structures; (2) align performance systems to promote cross-functional collaboration; and (3) implement supportive facilitation mechanisms to maintain momentum. These insights contribute to a more actionable application of ROT and offer generalizable lessons for hospital transformation in similarly fragmented or resource-constrained settings.

From the perspective of resource optimization and efficiency amelioration, the DEA method, integrated with real-world operational data, further provides empirical validation that the hospital operations management framework exerts a significant effect in mitigating inefficiencies within the Logistics Department. Through rational resource allocation and a dynamic distribution mechanism, the framework significantly enhances interdepartmental coordination and refines resource utilization rates. The implementation of this framework also ameliorates the rapid response capabilities of resource allocation, ensuring the hospital can achieve dynamic resource adjustments in the short term while promoting long-term sustainable development. This optimization process not only upgraded resource utilization efficiency but also strengthened organizational collaboration, further driving improvements in hospital operational efficiency. These findings align with prior research on intelligent hospital management models (Mi *et al*., 2023), which highlighted that the integration of AI and digital technologies enables more precise resource allocation. The implementation of automated systems serves to reduce the workload of medical staff while concurrently enhancing the hospital's managerial capabilities. This aligns with the principles outlined in the DEA analysis, emphasizing rapid response and dynamic resource distribution mechanisms, thereby providing technological support for improving hospital operational efficiency.

Enhancing patient experience constitutes another notable outcome of the framework's implementation. Through process optimization and the strengthening of cross-departmental collaboration, the hospital has achieved concurrent improvements in patient safety assurance, reduction in treatment duration and enhancement of medical service quality. Analysis of charts and data indicates that the framework significantly enhanced the hospital's social impact and competitiveness, along with a substantial rise in patient satisfaction. These enhancements enabled the hospital to better respond to external competitive pressures and expand its market share within the context of healthcare reform. The findings of this study regarding patient experience optimization are consistent with existing research on preventive interventions targeting unplanned hospitalizations. The latter highlights those key factors in improving patient experience, particularly in managing complex chronic diseases, including the integration of digital tools and collaborative platforms, which have been shown to be effective in increasing patient satisfaction (Herranz *et al*., 2024).

Additionally, the theoretical contributions of the framework have been explored. It demonstrates the potential applicability of the ROT in highly complex institutions, aligning with recent research that highlights the effectiveness of change management in complex hospital environments, which depends on optimizing centralization and information flow (Yousefi *et al*., 2022). Incorporating the DEA methodology, the hospital operations management framework provides a practical solution for resource optimization, effectively supporting sustainability and operational efficiency in hospitals.

The limitations inherent in the hospital operations management framework warrant attention. Notably, the implementation of this framework is confronted with challenges, including steep learning curves, interdepartmental frictions and staff adaptability deficits, which necessitate the enhancement of training initiatives for members of the Strategic Planning Group, Business Performance Group and Operations Management Group. Additionally, the framework lacks focus on critical factors influencing implementation outcomes, like digital transformation. Finally, its applicability across diverse healthcare settings remains unverified and demands further exploration, including variations in national contexts, regional settings (rural vs. urban) and hospital characteristics (public/private, scale). Previous research has involved the applicability of the framework across countries and regions. In high-income countries, technology-driven precision management is more suitable, leveraging technological advantages to enhance management efficiency (Hübner *et al*., 2024). Conversely, in low- and middle-income countries, simplifying the framework and focusing on optimizing key resource allocation may represent a more feasible approach (Twea *et al*., 2020). This consideration of cross-cultural and cross-economic contexts provides robust support for the global adoption of the hospital operations management framework. Addressing these challenges by exploring the framework's applicability in specialized hospitals and public health institutions would be a valuable focus for future research. Additionally, integrating emerging technologies such as artificial intelligence could further optimize collaborative network models and enhance overall management efficiency.

Although the hospital operations management framework proposed in this study has demonstrated significant effectiveness in enhancing collaboration efficiency, resource optimization and patient experience, a notable tension exists between its universal applicability and practical implementation. To maximize the framework's value, the following balancing relationships require dialectical examination:

First, the core principles of the framework – including interdepartmental collaboration incentive mechanisms, DEA-driven dynamic resource allocation and patient-centered process re-engineering – possess cross-scenario value. By eliminating information silos and optimizing decision-making processes, these principles provide a generalized methodology for efficient hospital operations. However, the framework's standardized processes face adaptability challenges in heterogeneous healthcare settings. Divergences in goal priorities between public and private hospitals, resource disparities between urban and rural institutions, and differences between specialized and general hospitals (e.g. oncology-specific hospitals prioritize bed turnover, while community hospitals focus on chronic disease management) necessitate highly contextualized implementation pathways.

Second, digital technologies (e.g. AI prediction, IoT-based scheduling) can significantly amplify the framework's effectiveness, achieving measurable improvements in resource utilization efficiency. Automated systems reduce administrative workload for medical staff, supporting the upgrading of managerial capabilities. While high-income countries can leverage technological advantages to advance precision management, low-income regions face a “technology applicability gap.” The framework's unstratified dependence on digital transformation risks exacerbating resource inequality, as its technical requirements are not tailored to varying resource endowments.

Finally, the current validation of the framework is confined to general hospitals (as in this study), and its applicability to specialized hospitals and public health institutions remains untested (e.g. privacy compliance constraints in psychiatric hospitals). Future research should (1) conduct multi-center contextual validation (e.g. rural medical alliances, private maternity hospitals): to assess marginal effects across heterogeneous settings; (2) explore cross-cultural adaptation factors (e.g. strengthening middle-management empowerment in high-power-distance cultures); and (3) integrate policy variables (e.g. the need to iterate resource optimization modules under case-based hospital payment reforms).

In conclusion, the hospital operations management framework proposed in this study provides both a valuable theoretical perspective and practical management strategies for hospital operations. Its broad applicability highlights its potential for widespread adoption in healthcare management.

## 6. Conclusion

The findings indicate that the implementation of the hospital operations management framework significantly refined overall operational efficiency, particularly by enhancing interdepartmental collaboration, optimizing resource allocation and improving patient experience. The study demonstrates that the cross-departmental incentive mechanisms helped overcome traditional information silos, enhance information flow and resource integration. This directly increased management efficiency, improved patient and staff satisfaction, and promoted the hospital's high-quality development.

The results also show that the framework significantly improved the resource utilization and operational efficiency through data-driven decision-making tools, such as the DEA method. The enabling rational resource allocation and dynamic response mechanisms enhanced short-term operational efficiency and laid a solid foundation for long-term sustainable development.

Furthermore, an outcome of the framework's implementation was the improvement in patient experience. The optimization processes and strengthening cross-departmental collaboration ensured patient safety while reducing treatment times and improving the quality of medical services. These enhancements provided the hospital with a competitive edge in external markets, expanded its social impact and strengthened its market competitiveness.

In summary, the hospital operations management framework proposed in this study not only provides valuable insights for theoretical research but also offers practical strategies for hospital management, showcasing broad application prospects. Future research should focus on addressing the learning curve challenges during the initial implementation phase and integrating emerging technologies, such as artificial intelligence, to further restructure collaborative network models and enhance overall management efficiency.
